# The function of histone methylation and acetylation regulators in GBM pathophysiology

**DOI:** 10.3389/fonc.2023.1144184

**Published:** 2023-05-02

**Authors:** Colin McCornack, Timothy Woodiwiss, Angela Hardi, Hiroko Yano, Albert H. Kim

**Affiliations:** ^1^ Medical Scientist Training Program, Washington University School of Medicine, St. Louis, MO, United States; ^2^ Department of Neurological Surgery, Washington University School of Medicine, St. Louis, MO, United States; ^3^ Department of Neurosurgery, University of Iowa Carver College of Medicine, Iowa, IA, United States; ^4^ Bernard Becker Medical Library, Washington University School of Medicine, St. Louis, MO, United States; ^5^ The Brain Tumor Center, Siteman Cancer Center, Washington University School of Medicine, St. Louis, MO, United States

**Keywords:** glioblastoma, histone postranslational modifications, histone post translational modifications, histone acetylation, histone methylation, glioblastoma epigenomics

## Abstract

Glioblastoma (GBM) is the most common and lethal primary brain malignancy and is characterized by a high degree of intra and intertumor cellular heterogeneity, a starkly immunosuppressive tumor microenvironment, and nearly universal recurrence. The application of various genomic approaches has allowed us to understand the core molecular signatures, transcriptional states, and DNA methylation patterns that define GBM. Histone posttranslational modifications (PTMs) have been shown to influence oncogenesis in a variety of malignancies, including other forms of glioma, yet comparatively less effort has been placed on understanding the transcriptional impact and regulation of histone PTMs in the context of GBM. In this review we discuss work that investigates the role of histone acetylating and methylating enzymes in GBM pathogenesis, as well as the effects of targeted inhibition of these enzymes. We then synthesize broader genomic and epigenomic approaches to understand the influence of histone PTMs on chromatin architecture and transcription within GBM and finally, explore the limitations of current research in this field before proposing future directions for this area of research.

## 1 Introduction

Glioblastoma (GBM) is the most common primary malignant brain tumor of the central nervous system with discouraging patient survival despite extensive research and clinical efforts to better understand and treat this malignancy. The median survival of 17-20 months in newly diagnosed GBM patients treated with standard of care has changed only modestly since the advent of the Stupp protocol published nearly two decades ago (1, 2). Although numerous clinical trials have been undertaken to improve outcomes in this disease, the standard of care for newly diagnosed disease—a combination of maximally safe resection, radiation therapy, and chemotherapy—has remained relatively unchanged for many years (2–4). Challenges to clinical progress include an incomplete understanding of cancer biology, a heterogeneous genetic and cellular environment, an immunosuppressive tumor microenvironment, and a delicate and difficult to access host organ system (5, 6). Extensive efforts have led to better characterization of the genetic and transcriptomic alterations in this cancer, but our understanding of the epigenetic regulation of this disease remains incomplete. Posttranslational modifications of histones play an important role in influencing transcription. Histone post-translational modifications (PTMs) have proven important in other forms of glioma, such as diffuse midline glioma, which commonly contain mutations in H3-K27M leading to global reduction in H3K27 methylation and increased PRC2-mediated repression of neurodevelopmental genes, potentially leading to lineage restriction and a preponderance of oligodendrocytic precursor like cells (7, 8). Similarly, the presence of IDH mutations in lower grade astrocytoma impacts the function of DNA methyltransferases and histone methyltransferases, leading to alterations in levels of activating and repressive histone post-translational modifications, as reviewed elsewhere (9). Methylation of the O6-Methylguanine-DNA methyltransferase (MGMT) promoter, which is associated with improved chemotherapy response, is a widely recognized epigenetic determinant in GBM, yet beyond DNA methylation, there is now a greater appreciation for the complex role that histone post-translational modifying enzymes play in regulating GBM pathophysiology (10, 11). This diverse group of enzymes carry out their effects *via* the modification of histone and non-histone substrates to control the ability of GBM cells to proliferate, invade surrounding tissue, and modulate the host immune response (12–14).

Epigenetics refers to heritable phenotypic changes that are independent of changes to underlying DNA sequences. These changes typically involve alterations in chromatin, a complex of the double-stranded DNA and an octamer containing two copies of the histone proteins H2A, H2B, H3, and H4. There are numerous PTMs that can be applied to the N-terminal tails and the core globular domains of these histone proteins, including, among other modifications, acetylation, methylation, and phosphorylation. Histone tail PTMs have varying impacts on the histone protein–DNA interaction, creating regions of transcriptionally-accessible chromatin (euchromatin) and transcriptionally-inaccessible chromatin (heterochromatin), which ultimately regulate functions such as transcription, DNA repair, and recombination. Importantly, recent efforts have suggested the ability of heterochromatin domains to persist through cellular division, thus representing a heritable aspect of information independent of DNA sequence identity (15, 16). Individual histone PTMs are associated with different states of transcriptional activation and repression and play a significant role in the broader landscape of the transcriptional machinery of a cell. Given the importance of transcriptional regulation, there are a variety of enzymes involved in regulating the modification of histone tails, including histone acetyltransferases (HATs/KATs), deacetylases (HDACs), methyltransferases (HMTs/KMTs), demethylases (HDMs/KDMs), ubiquitinases (ubiquitin ligases)/deubiquitinases, and protein kinases/phosphatases, which dynamically regulate the histone PTM landscape. In addition to their role in histone modification, many of these enzymes can modify non-histone substrates, including p53 demethylation *via* KDM1A, PRMT5-mediated arginine methylation of components of the ribonucleoprotein-assembling Survival of Motor Neurons (SMN) complex, and EZH2-mediated methylation and activation of STAT3 (17–19). Due to the diversity and complexity of these enzyme families, it has become increasingly important to understand their respective functions in the context of normal physiology and the impact of their dysregulation in human disease.

Histone PTMs at the global and local levels are frequently dysregulated in cancer, and the enzymes involved in histone modification have therefore become viable therapeutic targets. Large-scale genomic sequencing efforts have illuminated recurrent mutations of histone modifying genes in many distinct forms of cancer (20). These include *MLL*, *EP300*, and *CREBBP* in small cell lung cancer, *EHMT1* and *KDM6A* in medulloblastoma, and *EZH2* in diffuse large B cell lymphoma and follicular lymphoma (21–23). In addition to somatic mutations, histone modifying genes are often found to be over- or underexpressed in the context of cancer, such as EZH2 overexpression in prostate, bladder, ovarian, and breast cancer, MLL1 overexpression in colon cancer, and SIRT1 overexpression in prostate and colon cancer and downregulation in breast cancer and hepatic cell carcinoma (24–29). Although much of the research in GBM epigenetics and epigenomics has focused on DNA methylation, parallel research has shown alterations in the expression of histone modifying enzymes and the landscape of histone PTMs in primary GBM tumors (5, 30). Moreover, the well-established presence of GBM stem cells (GSCs) within primary tumors, along with the substantial transcriptional heterogeneity and plasticity found within GBM, raises a number of questions regarding whether histone PTMs and chromatin architecture play a role in regulating transcription and degree of differentiation, as exemplified by the role of specific histone demethylases in treatment escape in GSCs exposed to prolonged receptor tyrosine kinase inhibition (6, 31–36). In this review, we will discuss the current landscape of research into the role of histone modifying enzymes in GBM pathophysiology, highlighting research into histone tail acetylation and methylation enzymes, broader genomic characterizations of the histone landscape, and identifying the challenges and opportunities within this field of research.

## 2 Histone acetylation

The addition of acetyl groups to histone N-terminal domain lysine residues is catalyzed by the action of lysine acetyltransferases/histone acetyltransferases (KATs/HATs) whereas the removal of acetyl groups is catalyzed by histone deacetylases (HDACs). The HAT family of enzymes can be divided into subgroups based on structural and sequence homology — the Gcn5-related N-acetyl transferase (GNAT) family, the MOZ, Ybf2-Sas3, Sas2, and Tip60 (MYST) family, and the CBP/p300 family (37). These subgroups vary in specificity and roles outside of histone acetylation, such as the catalysis of p53 acetylation mediated by CBP/p300 and GCN5/PCAF or introduction of histone acetylation to regulate the binding of 53BP1 and influence DNA damage repair pathway selection (38, 39). HDACs are divided into four classes (Classes I–IV) based on similarity to yeast orthologs: Class I, comprised of HDAC1, HDAC2, HDAC3, and HDAC8; Class IIA, comprised of HDAC4, HDAC5, HDAC7, and HDAC9; Class IIB, comprised of HDAC6 and HDAC10; Class III, comprised of the Sirtuins 1-7; and Class IV, comprised solely of HDAC11 (40, 41). Of these, Classes I, II, and IV are Zn^2+^-dependent, whereas Class III/Sirtuins are NAD^+^-dependent. Like HATs, the enzymatic role of HDACs is not confined to histone deacetylation, with high levels of substrate promiscuity in the Sirtuin class of enzymes (40). Histone tail acetylation weakens the DNA-histone interaction by neutralizing the basic charge of the lysine residue, leading to decreased nucleosome occupancy and increased accessibility for RNA polymerase II binding and is often enriched at enhancers and promoters and correlated with transcriptional activity (42, 43). While this supports the view of HATs being associated with active genes and HDACs associated with inactive genes, genome-wide characterizations of HAT and HDAC activity reveal a more dynamic and nuanced picture, with HDACs serving roles in regulating active transcription as well as potentiating genes for future transcription (44). Given their functional role in both transcriptional regulation and modifications of non-histone substrates, HATs and HDACs have been implicated in many different disease states, including inflammatory diseases due to HAT-mediated post-translational modification of NF-κB, HAT-mediated acetylation of tau and concomitant increased expression of phosphorylated tau in Alzheimer’s disease, as well as overexpression, underexpression, and/or mutation of both HATs and HDACs in many different forms of cancer, as reviewed elsewhere (20, 40, 45–49). As diametrically opposed regulators of histone acetylation, the diverse families of HATs and HDACs play an important role in influencing the interaction between DNA and histones and consequently are significant actors in disease pathophysiology when this process becomes dysregulated.

### 2.1 Histone deacetylases and histone acetyltransferases in GBM

Like many other forms of cancer, the expression of HATs and HDACs is altered in GBM. Though comparatively less work has been done on the role of lysine acetyltransferases in GBM, expression of the lysine acetyltransferase KAT6A is upregulated in GBM, and its acetyltransferase activity promotes tumorigenesis through the regulation of *PIK3CA* expression and PI3K/AKT pathway activation (50). Early gene profiling experiments into the expression of HDACs and Sirtuins in GBM found significant decreases in *HDAC5* and *HDAC11* expression and significant increases in *HDAC6*, *HDAC7*, and *HDAC10* expression when compared to normal brain tissue (51). Further investigation of individual HDACs has begun to shed light on the role and function they play in the pathophysiology of GBM, and a catalog of this information can be found in **Table 1**. HDAC1 expression is elevated in GBM tumor tissue as compared to normal surrounding brain tissue, and early gene expression profiling experiments in GBM revealed TCGA subtype exclusivity in the activity of histone acetyltransferase and deacetylase pathways, with proneural tumors having increased activation of HDAC1 and mesenchymal tumors having increased activation of HDAC4 and SIRT1 pathways (51–53, 55). Further research into the functional consequences of HDAC1 knockdown in GBM cell lines revealed increased apoptosis and decreased cellular migration upon HDAC1 knockdown *in vitro*, alongside concomitant decreased levels of active AKT and ERK, highlighting a potential relationship between HDAC1 and the PI3K/AKT and Ras/ERK signaling pathways in GBM (53, 54). Additionally, selective inhibition of HDAC1 and HDAC3, enzymes whose expression in tumors is associated with significant decreases in overall survival of human GBM patients, leads to increased temozolomide (TMZ)-induced cell death *in vitro* through the hyperacetylation of the NF-κB subunit p65 and inhibition of its interaction with NF-κB coactivators KAT2B and KAT3B and increased interaction with ING4, a tumor suppressor (51, 56). In addition to its correlated pathway activation in mesenchymal tumors, HDAC4 overexpression led to increased cell proliferation, decreased reactive oxygen species (ROS) production, and increased invasiveness of U251 cells *in vitro*, and HDAC4 knockdown in U87 cells *in vitro* induced the expression of p21^WAF1/Cip1^, a cyclin-dependent kinase inhibitor and tumor suppressor involved in cell cycle regulation (55, 57, 58). Moreover, HDAC4 knockdown in U87 and U251 GBM cell lines led to radiation-induced senescence mediated by p21^WAF1/CIP1^ in addition to reducing neurosphere formation and the frequency of CD133^+^ and Nestin^+^ (stem) cells (59). A multivariate retrospective immunohistochemical analysis of GBM tumor tissue for HDAC4 and HDAC6 expression found that at the mean of the covariates, high expression of either or both HDACs is associated with decreased overall survival, conflicting with the findings of the cohort in a study by Dali-Youcef et al. (51, 59) Alongside these findings, HDAC6 knockdown was found to increase apoptotic cell death and autophagy in U251 GBM cells *in vitro*, with another investigation showing impaired EGFR pathway activation in HDAC6 knockdown U87 cells (60). A similar pathway dependency was found following knockdown of HDAC9, which led to reduced proliferation of U87 cells *in vitro*, potentially through downregulation of the EGFR/AKT/ERK pathway (61). Within the Sirtuin family of deacetylases, SIRT1 has been found to be associated with tumorigenesis and stemness in NSCs and GSCs, respectively, with selective inhibition of SIRT1 leading to increased p53-dependent transcriptional activity, acetylation, and apoptosis in NSCs but not in U87 cells (62). Additionally, SIRT1 expression decreases during differentiation of GSCs, along with their susceptibility to apoptosis *via* SIRT1 inhibition (62). There is conflicting evidence regarding the role and expression of SIRT2 in GBM. Early proteomic-based analysis found decreased SIRT2 expression in GBM tissue samples, and overexpression in GBM cell lines suppressed cell growth and induced changes in microtubule localization in one of the cell lines studied (63). Treatment with the polyphenol resveratrol led to SIRT2-mediated decreases in GSC proliferation. However, SIRT2 was also found to be expressed in GSCs but not NSCs (64). SIRT3, which is localized in mitochondria, is overexpressed in GSCs and plays an important role in GSC stemness and survival through its direct interaction with TRAP1, which together contribute to regulation of ROS primarily through the deacetylation of SOD2 (65). SIRT6 overexpression in T98G cells led to apoptosis and downregulation of the JAK2/STAT3 signaling pathway *in vitro*, though its influence on oncogenesis is cell-context dependent, and evidence regarding the relative expression of *SIRT6* in GBM is inconsistent (51, 66–68). While the role and dysregulation of HATs in GBM still requires targeted investigation, the altered expression of histone deacetylases in GBM has been found to have important functional consequences on stemness, tumorgenicity, and cell signaling, and thus these enzymes represent potential targets for treatment.

**Table 1:** The function of histone acetyltransferases & histone deacetylases in GBM.

Enzyme	Associated role in GBM	Cell lines/model system used	References
**KAT6A**	Promotion of H3K23 acetylation through interaction with TRIM24, leading to PI3K/AKT pathway upregulation	U87, LN229	(50)
**HDAC1**	Elevated expression in GBM; increased apoptosis, decreased cellular migration, and decreased MAPK signaling upon knockdown *in vitro*	Patient-derived cell cultures (52); U251, T98G (53); U87 (54)	(51–55)
**HDAC3**	Overexpression associated with decreased overall survival; inhibition leads to increased TMZ-induced cell death		(51, 56)
**HDAC4**	Overexpression associated with increased cell proliferation/invasiveness and decreased ROS production, knockdown associated with p21WAF/Cip1-mediated radiation-induced senescence and decreased stem marker expression	U87, U251	(55, 57–59)
**HDAC6**	Conflicting associations between expression in tumors and overall survival, knockdown impairs EGFR pathway and increases apoptosis and autophagy *in vitro*	U87, U251	(51, 59, 60)
**HDAC9**	Knockdown leads to reduced proliferation and downregulation of EGFR signaling pathway	U87	(61)
**SIRT1**	Selective inhibition leads to apoptosis in engineered NSCs and GSCs but not U87 cells, and reduced expression of stem markers in GSCs	U87, engineered NSCs, GSCs	(62)
**SIRT1**	Conflicting evidence – research on primary tumor tissue showed decreased protein expression and that overexpression in GBM cell lines suppressed cell growth, while contrasting research showed SIRT2 essentiality in mediating decreased cellular proliferation in GSCs upon treatment with resveratrol	Glioma cell lines (unspecified), GSCs, NSCs	(63, 64)
**SIRT3**	Overexpressed in GSCs, interacts with TRAP1 to activate SOD2 and prevent ROS overproduction. Knockdown leads to increased ROS production and loss of stemness	GSCs	(65)
**SIRT6**	Overexpression leads to apoptosis and JAK-STAT pathway downregulation *in vitro*, conflicting evidence about expression in GBM	T98G	(51, 66–68)

A summary of the functions of individual histone deacetylase and acetyltransferase enzymes in GBM pathophysiology, and the corresponding model system(s) used and reference to the original publication(s).

### 2.2 Histone deacetylase inhibition in GBM

In addition to targeted approaches assessing the role of histone acetylation-modifying enzymes in GBM, there has been increasing interest in the use of existing histone deacetylase inhibitors to treat GBM. A catalog of the inhibitors presented here can be found in **Table 2**. In addition to its anticonvulsant properties, valproic acid inhibits the activity of class I and II HDACs and has been demonstrated to augment radiation therapy in anti-cancer treatment (69, 80, 81). GBM-specific investigations have shown valproic acid treatment *in vitro* causes increased p21 ^WAF1/Cip1^ expression in multiple cell lines and sensitization to TMZ treatment in GBM cell lines but not in primary GSC cultures derived from human tumors (70, 71). Vorinostat (suberoylanilide hydroxamic acid, SAHA), a selective inhibitor of HDACs 1, 2, 3, and 6, has been used to treat certain types of cutaneous T cell lymphoma and has demonstrated similar ability to radiosensitize patient-derived GBM cell cultures *in vitro*, with this effect acting synergistically with a Bcl-2 pathway inhibitor, obatoclax (72–75). U87 cells treated with vorinostat exhibited cell cycle arrest in G_0_/G_1_ and reduced cell motility, whereas other studies have reported conflicting results for these phenotypes in patient-derived GBM cell lines (75, 76). In addition to these phenotypic changes, treatment with vorinostat was found to significantly alter the transcriptomic landscape of patient-derived GBM cell lines, with gene expression profiling revealing a shift away from TCGA proneural and classical molecular signatures towards a neural signature, though the existence of this particular molecular subtype has been questioned in more recent work (76, 82, 83). Trichostatin A, a class I and II HDAC inhibitor, has shown a similar ability to radiosensitize U87 and U373 cells *in vitro* and triggers similar transcriptional shifts away from TCGA proneural and classical expressional signatures in patient-derived cell lines *in vitro* (76, 77). Trichostatin A treatment of U251 and Hs683 cell lines *in vitro* has also been shown to upregulate mRNA expression of DIRAS-1, a small Ras GTPase and potential tumor suppressor in various solid tumors (84). Panobinostat, a nonselective HDAC inhibitor that has been explored as a potential therapeutic agent in a variety of cancers, has been shown to impact GBM cells in a manner similar to other HDAC inhibitors. Panobinostat radiosensitizes patient-derived GBM cell lines *in vitro*, with a greater effect on cell lines with *MGMT* promoter methylation (73). Panobinostat treatment in KLF9-overexpressing primary GBM neurospheres led to induction of apoptosis and necroptosis pathways *in vitro* (78). While the mechanism behind reduced cellular viability with treatment is undoubtedly multifactorial, *in vitro* and *in vivo* work by Nguyen et al. established a partial role for panobinostat-mediated disruption of c-Myc and subsequent metabolic shift to oxidative phosphorylation (79). Although HDACs represent a wide variety of enzymes with diverse downstream effectors, selective and broad inhibition of their function results in varying anti-tumor effects in GBM, including radiosensitization, sensitization to TMZ, and induction of cell death pathways. While the current preclinical evidence regarding HDAC inhibition in GBM is encouraging, clinical trials with the current generation of HDAC inhibitors have shown a mixture of outcomes with modest benefit in some trials and disappointing results in others due to unanticipated toxicity or failure to fill study arms (85). With newer therapeutic agents continually being generated, HDAC inhibition will undoubtedly continue to serve as a salient target for clinical trials for GBM (86–89).

**Table 2:** The effect of select HDAC inhibitors in GBM pathophysiology.

Treatment/Drug	Effect	Cell model(s) used	References
**Valproic acid**	*In vitro* radiosensitization of GBM cells, increased p21 expression in GBM model cells but not patient-derived cell lines	U87, T98G, TP365MG, U118MG, U251MG, U373MG, patient derived GSC lines	(69–71)
**Suberanilohydroxamic acid (SAHA, vorinostat)**	*In vitro* radiosensitization (compounded with concomitant Bcl-2 inhibition), leads to cell cycle arrest in G0/G1, shifts transcriptional phenotype away from proneural and classical transcriptional signatures	U87, GSCs	(72–75)
**Trichostatin A**	*In vitro* radiosensitization, shifts transcriptional phenotype away from proneural and classical transcriptional signatures, upregulates DIRAS-1 expression	U87, U373, U251, Hs683	(75–77)
**Panobinostat**	*In vitro* radiosensitization, apoptosis and necroptosis in neurospheres with concomitant KLF9 overexpression, metabolic shift to oxidative phosphorylation	GSCs/neurospheres, NCH644, NCH421k, U87	(69, 78, 79)

A summary of the impact of select HDAC inhibitors on GBM pathophysiology, and the corresponding model system(s) used and reference to the original publication(s).

## 3 Histone methylation

Histone tails are methylated through the action of histone methyltransferases, which catalyze the donation of methyl groups from S-adenosylmethionine to basic residues of the histone tail, whereas the removal of this modification is catalyzed by histone demethylases. Histone methylation most commonly occurs on lysine and arginine residues; lysine can be mono-, di-, or trimethylated, and arginine can be mono- or dimethylated, with dimethylation occurring either symmetrically or asymmetrically (90). Histone methyltransferases can be divided into three groups: SET-domain proteins and DOT1-like proteins (KMTs) which methylate lysine, and arginine *N*-methyltransferase proteins (PRMTs) which methylate arginine. Histone lysine demethylation is catalyzed by amine oxidase domain-containing proteins and Jumonji C (JmjC)-domain containing proteins, whereas the identification of selective arginine demethylases has proved elusive, with recent evidence suggesting dual lysine/arginine demethylase activity of certain lysine demethylase enzymes *in vitro* (91–93). Despite a few exceptions, these enzymes typically have higher substrate specificity compared to acetyltransferases, with specificity for unique methylation locations and degree of methylation (93–95). Unlike histone acetylation, the addition of methyl groups to histone tails does not result in charge neutralization of the target residue, instead altering the hydrophobicity and hydrogen bonding radius in the case of methyl-lysine, and thus the binding properties of these sites (96). Histone lysine methylation has a variety of correlations with transcriptional regulation and chromatin structure, depending on the location and degree of methylation. This includes associations of H3K4me1 with enhancer regions, H3K4me2 and H3K4me3 with promoter regions and transcription start sites, H3K27me3 with repressed transcriptional regions, and H3K36me3 in gene bodies of actively transcribed genes (44, 97, 98). Histone arginine methylation has been demonstrated to play a similarly important role in regulating transcription and chromatin architecture. Examples of this include the association of H3R2 symmetric dimethylation (H3R2me2s) with euchromatic promoters and H3K4me3 modifications, asymmetric H3R2 dimethylation (H3R2me2a) with promoter heterochromatinization, H4R3me2s with transcriptional repression and recruitment of DNMT3A, and CARM1 mediated methylation of H3R17 and H3R26 with transcriptional activation (99–103). However, histone methylation is context-dependent, as in the case of H3K4 methylation, where the plant homeodomain (PHD)-containing proteins recruited by this modification have varying functions in transcriptional activation and repression (96). Moreover, the colocalization of different methylation marks can lead to unique functions, as in the case of “bivalent” chromatin domains, such as embryonic stem cell transcription start sites marked by both H3K4me3 and H3K27me3, with loss of the repressive or activating mark during differentiation dependent on expression of the corresponding gene (104).

### 3.1 Histone demethylases in GBM

Research into the KDM (lysine-specific demethylase) family in GBM has provided insights into how these enzymes affect tumorigenicity through their dual role in demethylation of histone and non-histone substrates, a comprehensive summary of which can be found in **Table 3**. KDM1A (LSD1) is a H3K4/H3K9 demethylase that is overexpressed in GBM, which is consistent with similar overexpression in bladder, lung, and colorectal cancer (122). In GBM, expression in isolated GSCs is inversely correlated with degree of differentiation (105). Initial research on the function of KDM1A in GBM focused on the similarities in the catalytic domain between KDM1A and monoamine oxidases (MAOs), finding that inhibition of KDM1A with the MAO inhibitor tranylcypromine rendered GBM cell lines more sensitive to treatment with HDAC inhibitors, but this synergistic effect was not observed in immortalized human astrocytes (106). In addition, selective inhibition of KDM1A through small molecule inhibitors or shRNA has been shown to decrease cellular proliferation, colony formation, and *in vivo* tumor progression. In tandem with these changes, expression of stem cell-associated genes decreased, and expression of genes involved in the unfolded protein response pathway increased, partially mediated by increases in H3K4me2 at associated loci (105, 107). However, the work of Kozono et al. complicated the conclusion that KDM1A promotes tumorigenicity. Instead, their findings suggested a dose-dependent influence of KDM1A on tumorigenicity as partial inhibition was associated with increased H3K4me3 at the MYC locus, increased MYC expression, and increased downstream expression of stem cell-associated genes, whereas complete inhibition led to decreased MYC expression and consequent cell death (108). Subsequent work described a mechanism by which GSK3β increases KDM1A stability *via* phosphorylation, allowing for downstream increases in USP22-mediated deubiquitylation and H3K4 demethylation activity of KDM1A. In turn, increased KDM1A binding to BMP2, CDKN1A, and GATA6 promoters repressed transcription of these genes, while increasing the expression of stem cell-related genes (109). Loss of either of the H3K36 demethylases KDM2A or KDM2B resulted in dysregulation of several GBM cellular phenotypes. Knockdown of KDM2A, a target of the microRNA miR-366 which is downregulated in GBM, resulted in reduced cellular proliferation, migration, and invasiveness (110). Alongside similar reductions in cellular viability, KDM2B knockdown reduced GSC self-renewal and increased sensitization to chemotherapy treatment with the alkylating agent lomustine, alongside increased susceptibility to TRAIL-induced apoptosis (111, 112). In addition to upregulation of mean expression in primary GBM tumor samples, the H3K9/H3K36 demethylase KDM4A has been shown to be upregulated in TMZ-resistant GSCs (113). KDM4A knockdown in GBM cell lines *in vitro* led to increased apoptosis and reductions in cellular viability and invasiveness, and these effects were ameliorated by the suppression of autophagy (114). A separate investigation suggested a connection between KDM4A and the mTOR pathway, a negative regulator of autophagy, with KDM4A overexpression and knockdown leading to increased and decreased activation of the mTOR pathway, respectively (115). Knockdown of the H3K9/H3K36 demethylase KDM4C led to a reduction in CD133^+^ GSCs and reduced cellular viability, potentially mediated by a link between KDM4C and c-Myc/p53, in which KDM4C demethylates p53 and inhibits its roles in transcriptional activation and initiation of apoptotic pathways (116, 117). KDM5A, a H3K4 demethylase, has been shown to be markedly elevated in TMZ-resistant GSCs, a finding that is consistent with similar overexpression seen in drug-resistant non-small cell lung cancer (123). Exogenous KDM5A overexpression inhibited TMZ-induced apoptosis in GBM cell lines (113). This finding was further supported by work showing significant decreases in cellular viability in TMZ-resistant subclones treated with the selective KDM5A inhibitor CPI 455 (118). Fellow H3K4 demethylase KDM5B has been found to have higher expression in GBM tumor tissue than normal surrounding brain tissue and has an inverse correlation with overall survival post-resection (119). Expression of H3K27 demethylase KDM6B is upregulated in TMZ-resistant GSCs, and selective inhibition of this enzyme *via* the drug GSK J4 led to reduction of cell cycle transition to G2 and induction of apoptosis, though these phenotypes did not differ between TMZ-naïve and TMZ-resistant populations (113, 120). However, the expression of KDM6B in human GBM tumors is variable and heterogeneous, and contrasting research has suggested that overexpression of this enzyme inhibits neurosphere formation *in vitro* and tumor formation *in vivo* and further that STAT3-mediated repression of KDM6B expression is essential for neurosphere formation and cellular proliferation (121). With some exceptions, the function of histone demethylases primarily acts to promote tumorigenicity, thus serving as a potential therapeutic target to abrogate proliferative and anti-apoptotic functions in GBM.

**Table 3:** The function of histone demethylases in GBM.

Enzyme	Associated role in GBM	Cell lines/model system used	References
**KDM1A**	Overexpressed in GBM, particularly in stem-like cells. Constant inhibition decreases proliferation, colony formation, and tumorgenicity, and sensitizes cells to HDAC inhibitors, while transient inhibition increases stem gene expression. Stabilized by GSK3β-mediated phosphorylation,	GSCs, U251, U87, SNB-19, LN-18	(105–109)
**KDM2A**	Knockdown associated with reduced proliferation, migration, and invasiveness	A172, U251, T98G	(110)
**KDM2B**	Knockdown associated with reductions in cellular viability and self-renewal, increased sensitization to CCNU, and increased susceptibility to TRAIL-induced apoptosis	Patient-derived cultures, U87, T98G	(111, 112)
**KDM4A**	Upregulated in TMZ-resistant GSCs, knockdown results in reduced mTOR pathway activation, reduced invasiveness, and autophagy-dependent apoptosis	A172, U87MG, T98G, U251	(113–115)
**KDM4C**	Knockdown leads to reduced cellular viability, KDM4C acts as a p53 demethylase to inhibit initiation of apoptotic pathways	GSCs, U87, U251	(116, 117)
**KDM5A**	Upregulated in TMZ-resistant GSCs, overexpression inhibits TMZ-induced apoptosis in GBM cell lines, inhibition in TMZ-resistant subclones leads to decreased cellular viability	A172, U251, CAS1, DBTRG, U87, GSCs	(113, 118)
**KDM5B**	Higher expression in tumor tissue than surrounding brain, expression inversely correlated with overall survival post-resection	SW1783, U-87, LN-18, Hs683, and T98G	(119)
**KDM6B**	Conflicting evidence: Upregulated in TMZ-resistant GSCs, inhibition has been shown to induce apoptosis in both TMZ-naïve and TMZ-resistant cells. Overexpression has been shown to inhibit neurosphere formation *in vitro* and *in vivo*, and STAT3-mediated repression causes normal neurosphere formation	A172, U251, DBTRG, GSCs, NSCs.	(113, 120, 121)

A summary of the functions of individual histone demethylase enzymes in GBM pathophysiology, and the corresponding model system(s) used and reference to the original publication(s).

### 3.2 Histone methyltransferases in GBM

Trimethylation of H3K27 is a ubiquitous repressive mark found in large stretches of heterochromatic DNA and is associated with transcriptional repression. The introduction of this modification is catalyzed by Polycomb Repressive Complex 2 (PRC2), a multiprotein complex that carries out methyltransferase enzymatic function *via* the enhancer of zeste homolog 2 (EZH2) subunit (124). Adding complexity to its regulatory role, EZH2 can methylate non-histone substrates within the nucleus or the cytosol (125). Aberrant EZH2 expression is a hallmark of many cancers and elevated expression in the context of malignancy can be a marker of poor prognosis and advanced disease (126). EZH2 expression has been shown to be elevated in GBM, and its expression is similarly correlated with a poorer prognosis (12, 127–129). Due to the importance of H3K27me3 in transcriptional regulation and chromatin architecture, extensive efforts have been made to understand the role of EZH2 in promoting GBM tumorigenicity. EZH2 has been shown to exert diverse regulatory roles in GBM, modulating pathways in tumor initiation/self-renewal, differentiation, cell cycle progression, metabolism, immunogenicity, and invasiveness. Early work by Suvà et al. showed that EZH2 is necessary for tumor formation and self-renewal in patient-derived GSCs, with further research highlighting the importance of an AKT-mediated interaction between EZH2 and STAT3 in GSC self-renewal (19, 32, 130–133). There is evidence that EZH2 mediates both pro- and inhibitory differentiation signals. One mode of inhibition of GSC differentiation occurs through hypermethylation of the BMPR1B promoter, thought to be mediated by EZH2 recruitment of DNMT1, allowing for clonal expansion *via* inhibition of differentiation (134). In contrast, differentiation is induced *via* H3K27me3-mediated suppression of Nanog (131). Investigations using a transgenic high-grade glioma mouse model demonstrated that FZD8, a G protein-coupled receptor involved in Wnt signaling, undergoes H3K27me3-mediated suppression during tumorigenesis and that this could be an early disruptor of normal differentiation pathways during gliomagenesis (135). Fitting with this theme, Mortimer et al. provided compelling evidence for redistribution of EZH2 binding sites across the genome following malignant transformation, most significantly at *HOX* genes (136). EZH2 inhibition has been shown to impact cell cycle progression, with inhibition leading to apoptosis and block of cell cycle progression in a p16, p21, and p27-mediated manner (12, 137–139). Alterations in metabolic pathways is a hallmark of cancer, and EZH2 has been shown to upregulate glycolysis *via* increased HIF1α expression, a known transcription factor important for activism of metabolism-related genes. EZH2 promotes the glycolysis pathway *via* binding to the promoter of a known HIF1α repressor EAF2, resulting in H3K27me3-mediated repression (128). A role for EZH2 in the regulation of fatty acid metabolism has been suggested by *in vitro* and *in vivo* knockdown of EZH2, which correlated with decreased lipid metabolism and decreased expression of PGC-1a, FASN, and SREBP-1. Interestingly, TERT appears to be a co-regulator of EZH2 in this pathway, demonstrating an ability to restrict the repair of DNA damage *via* downregulation of phospho-ATM, providing fitness/adaptation benefits through increased genomic instability (140). Further adding intrigue to EZH2 modulation of DNA damage repair, De Vries et al. showed that prolonged EZH2 inhibition in a syngeneic mouse model leads to enhanced tumor growth after an initial 3 week period of inhibited growth. This reversion to the pre-inhibited tumor growth state appears to be due to enhanced DNA damage repair within tumor cells (141). EZH2 also contributes to the immunosuppressive microenvironment of GBM by triggering specific cytokine expression, maintaining expression of interferon-stimulated genes that promote a M2 microglial phenotype in an iNOS and TGF-β2-dependent manner. Evasion of NK cell immune surveillance occurs *via* a circular EZH2 encoded protein (EZH2-92aa) that directly binds the promoters of genes (MICA/B, ULBP) necessary for the expression of NK group 2D ligands in GSCs, leading to decreased transcription and ultimately decreased NK cell-mediated tumor cell death (14, 139, 142, 143). The role of EZH2 in promoting GBM invasiveness *via* regulation of AXL in a histone modification-independent manner has been demonstrated *in vitro* with EZH2 knockdown (127). Expanding upon this work, another group showed that EZH2 inhibitors decrease invasiveness by downregulating VEGF, matrix metalloproteinases, and cell surface adhesion markers (E-cadherin and N-cadherin) (139). Several non-coding RNAs have been shown to be important in EZH2-mediated invasiveness. The lncRNA NEAT1, which is upregulated by EGFR, forms a scaffold with EZH2, which together augment invasion by increasing nuclear β-catenin. (144) This activation of β-catenin also appears to feedback on EZH2 activity by increasing expression of USP1, a deubiquitinase that stabilizes EZH2 (145). The microRNA, miR-490-3p, undergoes EZH2-mediated H3K27me3 silencing, resulting in increased colony formation and transwell migration *in vitro* (13). It is evident that EZH2 plays a broad and diverse role in the regulation of tumorigenicity in GBM tumor cells, highlighting its significant clinical potential as a therapeutic target.

The family of H3K9 methyltransferases has been shown to be similarly important in GBM tumorgenicity. Euchromatic histone lysine methyltransferase 2 (EHMT2), also known as G9a, mediates repressive mono- and dimethylation of H3K9 and its expression is associated with improved survival in grade II oligodendrogliomas. Contrasted with a protective role in oligodendrogliomas, early work on the role of EHMT2 in GBM tumorigenicity was mixed, but with more recent evidence supporting a pro-tumorigenic role (146, 147). *In vitro* studies have shown that inhibition of EHMT2 in established GBM cell lines promoted GBM cell growth and increased expression of stem cell markers, and direct methylation of HIF-1α by EHMT2 inhibits hypoxia adaptation and cellular invasion (148, 149). Conflicting evidence demonstrates that EHMT2 contributes to tumorigenicity in GBM. *In vitro* assays in established GBM lines show that EHMT2 is required for proliferation, migration, and invasion in a c-Myc dependent manner and that inhibition of EHMT2 leads to reduced global H3K9me2 and to increased apoptosis, autophagy markers, and differentiation in human primary GSCs (150, 151). Further work revealed EHMT2-mediated evasion of IFNγ-directed apoptosis and increased survival with EHMT2 knockdown in GSCs in an orthotopic nude mouse model (150, 152). Similar to EHMT2, Suv39H1 and SETDB1 decrease gene expression through their H3K9 methyltransferase activity (153, 154). Studies evaluating their role in the setting of GBM have found increased expression of both genes compared to normal brain, and decreased cell proliferation, increased apoptosis, reduced migration, and reduced colony formation upon shRNA knockdown of SETDB1 or inhibition of Suv39H1 with chaetocin in established GBM cell lines (155, 156). Interestingly, there appears to be a relationship between poor survival and increased cytoplasmic Suv39H1 that does not exist for nuclear Suv39H1, suggesting a histone independent mechanism of pathogenesis. Thus, there is a compelling role for EHMT2 in the promotion of GBM proliferative and invasion, which is consistent with its pro-malignancy role in numerous other cancers, yet further work in needed to fully understand its role in GBM (157). Similarly, further work is needed to characterize the mechanisms by which SETDB1 and Suv39H1 mediate the observed phenotypic changes as well as their impact on chromatin architecture and organization in GBM.

Due to the role of arginine methyltransferases enzymes in AML, melanoma, and lung cancer recent efforts have been made to characterize their role in GBM pathogenesis (133, 158–160). The arginine methyltransferase PRMT3, PRMT5, and PRMT6 have elevated expression in GBM tissue, and their expression is associated with decreased survival (161–163). In contrast to the pro-tumorigenic effect of these enzymes, PRMT1 plays an antiproliferative role by counteracting the effect of EHMT2 in the presence of IFNγ (152). PRMT3 appears to regulate multiple metabolic pathways in GBM with a specific role in preventing ubiquitination of HIF1α, thereby promoting glycolysis (163). PRMT3 knockdown in GSCs induced cell cycle arrest and apoptosis, and its inhibition led to decreased tumor growth in a nude mouse flank model (163). *In vitro* knockdown of PRMT5 reduced colony formation, migratory activity, and led to increased cell cycle arrest and apoptosis (161, 164, 165). Further work showed that PRMT5 downregulates PTEN *via* promoter binding and ultimately leads to increased active ERK and AKT (164). PRMT5 is also used by GBM cells to evade mTOR inhibition, and PRMT5 inhibition causes widespread disruption of mRNA splicing, especially in cell cycle related genes (166, 167). Adding validity to this *in vitro* work, inhibition of PRMT5 *in vivo* increased animal survival (164, 166, 167). Inhibition of PRMT6 limits RCC1 driven mitotic activity, leading to decreased tumor growth and increased radiation sensitivity *in vivo* (162). Overall, these studies provide an initial characterization of the function of arginine methyltransferases in GBM, but more work is needed to clarify their influence on genomic architecture and transcriptional regulation.

Much less is known about the various other human histone methyltransferases in the context of GBM. H3K4 methyltransferase KMT2A (MLL1) expression increases in GBM in the setting of hypoxia in a HIF-dependent manner, with knockdown leading to decreased self-renewal *in vitro* and decreased tumor formation *in vivo* (168). Although KMT2E (MLL5) has no catalytically active histone methylation domain, its expression is anticorrelated with H3K4me3 levels in primary GBM cultures, and knockout reduced self-renewal capacity (94, 169). DPY30 is the catalytic subdomain of the MLL/SET1 family of proteins, and recent work explored its role in GBM based on an RNAi screen demonstrating that DPY30 knockdown decreases cell viability *in vivo*. Interestingly, *in vitro* inhibition had no effect, which is consistent with the demonstrated pro-tumorigenic mechanism of DPY30 in GBM cells where it improves hypoxia adaptation and activates angiogenesis pathways (170). A subset of low-grade glioma and GBM patients harbor an inhibitory mutation in *SETD2*, and decreased SETD2 expression is associated with poor prognosis in GBM. Higher secretion of TGF-β1 in GBM cells derived from patients carrying the *SETD2* mutation led to an increase in activated tumor-associated microglia which fueled tumor progression (171). Additional work has shown that EGFR-mediated suppression of SETD2 results in decreased DNA damage repair, resulting in an accumulation of DNA damage in established GBM cells lines, leading to increased mutagenesis and subsequent selective adaptation (172). Stabilization of SETD2 with Palmostatin-B, a drug the prevents de-palmitoylation, led to decreased proliferation of established GBM cell lines and decreased tumor growth in a nude mouse model, consistent with an antiproliferative role for SETD2 in GBM (172). Comparison of periventricular human GBM to normal subventricular zone NSCs obtained from non-human primates suggested a potential role for the H4K20 methyltransferases KMT5B and KMT5C (Suv420H1/2) in GBM tumorigenesis, showing that 21-31% of genes repressed by the H4K20me3 mark in NSCs are upregulated in GBM cells (173). Finally, one study has shown that SMYD3, a member of the SMYD lysine methylase family, promotes proliferation and tumorigenicity in established GBM cell lines *in vitro* and *in vivo* (94, 119, 174). Despite the important role that the MLL/SET1 family of enzymes play in other cancers, less is known about these enzymes in the context of GBM and more work is needed to better elucidate their role in regulating and promoting tumorigenesis through their modifications of histone and non-histone substrates (175). A comprehensive summary of the functions of the methyltransferase enzymes described herein can be found in **Table 4**.

**Table 4:** The function of histone methyltransferases in GBM.

Enzyme	Associated role in GBM	Cell lines/model system used	References
**EZH2**	Overexpressed in GBM. Increases cell cycle progression, invasiveness, tumorigenicity, and tumor growth. Modulates metabolism, differentiation, and immune signaling.	A172, BCRC 60380, BCRC 60163, GL261, H4, LN18, LN229, N33, T98G, U87MG, U251, patient-derived GSCs, patient-derived neurospheres, nude mouse model, syngeneic mouse model	(12–14, 19, 32, 127, 128, 130, 132, 134–145, 176–186)
**EHMT2**	Promotes proliferation, migration, and invasion. Reduces differentiation, apoptosis, and autophagy.	A172, LN18, LN229, U87MG, U251MG, patient derived GBM cell cultures, patient derived GSCs, nude mouse model	(148–152, 187)
**Suv39H1**	Overexpressed in GBM. Promotes proliferation, migration, and colony formation.	T98G, U87MG	(155, 156)
**SETDB1**	Overexpressed in GBM. Promotes proliferation, migration, and colony formation.	T98G, U87MG	(155, 156)
**PRMT1**	Decreases GBM cell viability in a counter-regulatory fashion to EHMT2	A172, U87MG	(152)
**PRMT3**	Overexpressed in GBM. Regulates glycolysis and inhibition increases apoptosis, inhibitors cell cycle progression, decreases tumor growth	U87, U251, patient derived GSCs, nude mouse flank model	(163)
**PRMT5**	Overexpressed in GBM. Promotes colony formation, migration, and cell cycle progression.	LN229, U87EGFRvIII, patient derived GBM cell cultures, patient derived neurospheres, patient derived GSCs, zebrafish GBM model, nude mouse model	(161, 164, 166, 167)
**PRMT6**	Overexpressed in GBM. Promotes cell proliferation.	T98G, U87, patient derived GBM cells, nude mouse model	(162)
**MLL1**	Promotes self-renewal and tumor formation.	Patient derived GSCs, nude mouse model	(168)
**MLL5**	Promotes self-renewal	Patient derived GBM cell lines, nude mouse model	(169)
**SMYD3**	Promotes cell proliferation and tumorigenicity	HEB, LN18, T98G, U87, U373, nude mouse flank model	(188)
**DPY30**	Promotes cell viability through regulation of hypoxia and angiogenesis	Patient derived GBM cell lines, patient derived GSCs, nude mouse model	(170, 189)
**SETD2**	Antiproliferative effects are neutralized in GBM through mutation or EGFR suppression	Patient derived GBM cell lines, patient derived GSCs, nude mouse model	(171, 172)
**KMT5B/5C**	Dysregulation implicated in gliomagenesis	Baboon and mouse-derived NSCs	(173)

A summary of the functions of individual histone methytransferase enzymes in GBM pathophysiology, the associated histone post-translational modification, and the corresponding model system(s) used and reference to the original publication(s).

### 3.3 Inhibition of histone demethylases and methyltransferases in GBM

Numerous inhibitors have been used in the laboratory to better understand the role of histone methyltransferases and demethylases in GBM pathophysiology, with many shown to have anti-proliferative effects. A summary of these inhibitors can be found in **Table 5**. Inhibitors that target KDMs range from broad class inhibition to individual enzyme specificity. Examples of KDM inhibitors with broad enzymatic targets include dimethyloxaloglycine (DMOG), GSK-14, and JIB-04. DMOG has been shown to induce DNA damage and apoptosis in GSCs through targeting of the Jumonji (JMJ) family of demethylases (KDM2-KDM7) (116). Although GSK-14 is a broad KDM class inhibitor, its antiproliferative effects in GCSs appear to operate through inhibition of KDM2B (111). JIB-04 is another broad inhibitor of KDMs with some specificity for KDM5A and has been shown to activate autophagy and apoptosis in established GBM cells lines *in vitro* (118). Additional work demonstrated a synergistic effect when JIB-04 is combined with GSKJ4, a KDM6B inhibitor, in TMZ resistant cells *in vitro* (120). Several targeted inhibitors of KDM1A, KDM4C, and KDM6B have been investigated in GBM. The tricyclic antidepressant tranylcypromine, which also functions as a KDM1A inhibitor, caused apoptosis in established GBM cells lines when combined with vorinostat (106). NCL-1 and NCD-38 are small molecule inhibitors that target KDM1A and preferentially affect GSCs, leading to apoptosis *in vitro* and increased survival *in vivo*, without notable effects on differentiated cells (105). Similarly, selective inhibition of KMD4C by SD70 decreased cell viability *in vitro* in established GBM cell lines (117). In GSCs, inhibition of KDM6B *via* GSKJ4 inhibited cell growth through activation of apoptosis pathways (36, 120).

**Table 5:** The effect of select histone methyltransferase and histone demethylase inhibitors in GBM pathophysiology.

Treatment/Drug	Target	Effect	Cell model(s) used	References
**Tranylcypromine**	KDM1A	Increased cell death in combination with vorinostat	LN-18, U87	(106)
**NCL-1 & NCD-38**	KDM1A	Reduced viability and increased survival	GSCs, U251, murine mouse model	(105)
**GSK-14**	KDM class/KDM2B	Decreased cell viability	GSCs	(111)
**SD70**	KDM4C	Decreased cell viability	U251, U87	(117)
**JIB-04**	KDM class/KDM5A	Activated autophagy and apoptosis	A172, U251, GSCs	(118, 120)
**GSKJ4**	KDM6B	Decreased cell growth and increased apoptosis	U251, GSCs	(36, 120)
**DMOG**	KDMs 2-7	Induced DNA damage and apoptosis	GSCs	(116)
**AC1Q3QWB**	EZH2	Increased cell death and decreases tumor growth when combined with DZNep	N5, N33, murine flank model	(184)
**DZNep**	EZH2	Decreased self-renewal and tumor growth	U87, U251, LN229, D54, GSCs, murine mouse models	(12, 19, 131, 132, 181, 190)
**GSK126**	EZH2	Decreased pSTAT3	GSCs	(19)
**PCI-24781**	EZH2	Reduced proliferation and induced cell cycle arrest and apoptosis	LN18, LN229, U87	(137)
**UNC1999**	EZH2	Decrease cell viability, induced autophagy, reduce flank tumor growth	GSCs, murine mouse model	(179)
**EPZ6438**	EZH2	Increased apoptosis and survival	GSCs, murine mouse model	(135, 181)
**MC4040 & MC4041**	EZH2	Cell cycle arrest, decreased invasiveness	U87, patient derived cell cultures	(139)
**BIX01294**	EHMT2	Decreased self-renewal and cell viability, activation of autophagy, reduced tumor growth	U87, U251, LN18, LN229, D54, GSCs, murine mouse model	(148, 150, 151, 187, 190, 191)
**Chaetocin**	SUV39H1	Reduced proliferation and clongenic ability	T98G	(156)
**SGC707**	PRMT3	Inhibited cell growth and glycolysis, Inhibited tumor growth	U87, U251, patient derived GSCs, nude mouse flank model	(163)
**HLCL65, CMP12**	PRMT5	Increased cell death, improved survival	Patient derived cell cultures, zebrafish GBM model	(165)
**CMP5**	PRMT5	Inhibits self-renewal and cell cycle progression, increased apoptosis and survival, long term survivors	Patient derived cell cultures, zebrafish GBM model	(165)
**GSK591 & LLY-283**	PRMT5	Inhibits proliferation and sphere formation, increases apoptosis and survival, crosses BBB	GSCs, murine mouse model	(167)
**EPZ020411**	PRMT6	Induces cell cycle arrest, decreases sphere formation, increased survival	GSCs, murine mouse model	(162)

A summary of the impact of select histone methyltransferase and histone demethylase inhibitors on GBM pathophysiology, and the corresponding model system(s) used and reference to the original publication(s).

The majority of the work on HMT inhibitors has focused on EZH2 and to a lesser extent EHMT2, with more recent work focusing on arginine methyltransferases. The most studied inhibitor of EZH2 is 3-deazanoplanocin A (DZNep), with numerous studies demonstrating its ability to decrease GBM cell self-renewal and viability *in vitro* and decrease tumor growth *in vivo* (14, 19, 131, 132, 190, 192). *In vitro* and *in vivo* work in a murine flank tumor model demonstrated that AC1Q3QWB, a small molecule inhibitor of EZH2’s interaction with the lncRNA HOTAIR, increases cell death and decreases tumor growth (184). Similarly, the small molecule inhibitor EPZ6438 has been used to inhibit EZH2 in GSCs and murine GBM models, leading to increased apoptosis *in vitro* and increased survival *in vivo* (135, 181). EPZ6438 has also been shown to accumulate intracranially in murine tumors (181). In contrast, although targeting EZH2 with UNC1999 decreased GSC viability and self-renewal *in vitro*, *in vivo* studies did not show benefit in orthotopic xenografts despite decreased growth in flank models, suggesting low brain penetration. Further *in vitro* studies demonstrated reduced cell viability and increased apoptosis *via* inhibition of EZH2 with the small molecule inhibitors PCI-2478, MC4040, and MC4041 (137, 139, 179). Multiple studies have evaluated the role of BIX01294 as an EHMT2 inhibitor in GBM. *In vitro* experiments demonstrated decreased GBM cell viability *via* apoptosis and autophagy and increased GSC differentiation, in line with *in vivo* experiments which showed reduced tumorigenicity (62, 148, 150, 151, 187, 190). Finally, one study utilized chaetocin to inhibit SUV39H1, which reduced GBM cell clonogenic potential and migratory ability (156).

Multiple inhibitors of arginine methyltransferase enzymes PRMT3, PRMT5, and PRMT6 have shown promising results in recent years. Work in patient-derived GSCs and a nude mouse flank model demonstrated decreased glycolysis, cell growth, and tumor growth with SGC707, a small molecule PRMT3 inhibitor (163). A zebrafish GBM model was used to identify numerous inhibitors of PRMT5 with anti-proliferative effects. Three compounds (HLCL65, CMP12, CMP5) were identified, all of which provided *in vitro* cytotoxicity and increased survival *in vivo*, although CMP5 appeared most promising as treatment led to a significant number of long-term survivors (165). Further work targeting PRMT5 in GSCs showed that the compounds GSK591 and LLY-283 decreased *in vitro* proliferation and sphere-forming capacity with evidence of blood-brain barrier (BBB) drug penetration by LLY-283, leading to increased survival *in vivo (* 167 *).* The PRMT5 inhibitor, EPZ01566, in concert with mTOR inhibitors provided anti-proliferative effects *in vitro* and increased survival *in vivo* (166). A sole PRMT6 inhibitor (EPZ020411) has been shown in GSCs to induce differentiation and cell cycle arrest, and increased *in vivo* survival was most pronounced when combined with ionizing radiation (162). Overall, these numerous studies demonstrate the utility of using small molecule inhibitors to target histone modifying enzymes in GBM, but work investigating the brain penetration of these drugs or opportunities to combine these drugs with BBB modulating technologies, as well as studies characterizing their impact on non-GBM cells in the tumor micro-environment (TME) are needed.

## 4 Genomic landscape of GBM

**Figure 1:**
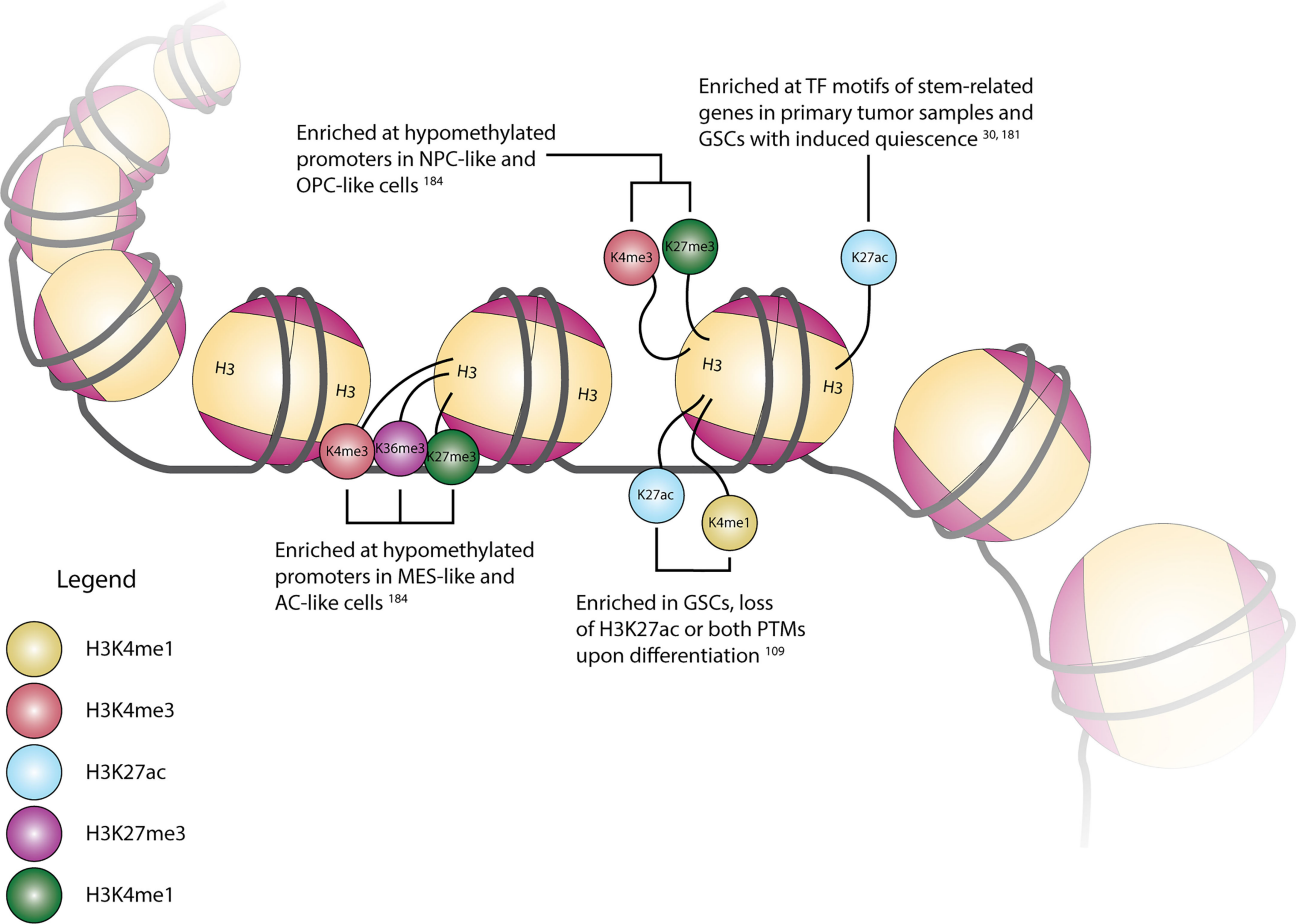
Cartoon depiction of common histone H3 modification locations and their associated modifications, transcriptional state associations, and/or regulation in GBM.

The potential functional impact of the diverse enzymes with roles in histone PTMs can be seen through the presence and location of these various alterations across the genome. A summary of selected modifications can be seen above in **Figure 1**. Early work in this area explored the conversion of GSCs to more terminally-differentiated brain tumor cells, a process dependent on PRC2-mediated H3K27me3 at the BMP5 locus and a concordant loss of this modification at the Wnt1 promoter (131). The essentiality of Wnt signaling in GSC maintenance was further underscored by the dual observation of increased expression of Wnt-pathway activator ASCL1 in GSCs and ASCL1 binding to a H3K4me1-marked poised enhancer of Wnt signaling inhibitor DKK1, preventing its expression (193). However, further research into the role of ASCL1 in promoting stemness or differentiation suggested that its role may be context-dependent, as separate work suggested that ASCL1 can independently direct GSCs to a neuronal fate, downregulate cell cycle genes *in vivo*, and act as a potential pioneer factor for neuronal target genes (194, 195). Loss of stem-like properties in GSC populations is also associated with global chromatin changes in H3K4me3/H3K27me3 (“bivalent”) histone modifications. Genome-wide profiling of H3K4me3 and H3K27me3 in eight GSC lines in comparison to human astrocytes revealed unique bivalent modifications at loci for a variety of gene families, including HOX family genes, Wnt pathway genes, Hedgehog signaling, and solute carrier family genes (196, 197). In comparison to fetal neural stem cells (fNSCs), roughly 37% of H3K4me3/H3K27me3 marks at promoter regions were found to be unique to GSCs, with 137 promoter regions containing this bivalent modification in fNSCs but only the H3K4me3 modification in GSCs, and 191 promoter regions containing both modifications in fNSCs but only the H3K27me3 modification in GSCs (198). A similar comparison of chromatin states between fNSCs and GSCs revealed that GSCs had lost brain-specific H3K4me1-marked active enhancers, as well as transitioning to poised or active enhancer marks in other tissue-specific enhancers. Additionally, GSC specific regions with colocalized H3K4me1 and H3K27ac marks were enriched for gene ontology terms related to angiogenesis and DNA damage response pathways (199). Upon repression of stem cell-like properties in GSCs, genes with histone mark changes from H3K4me3 to H3K27me3 included Wnt-signaling pathway mediator *LEF1*, and *ARNT2*, a mediator of the hypoxia response pathway involved in promoting the expression of stem cell markers *OLIG2*, *POU3F2*, and *SOX9* (200). Expanding upon the observation that primary GBM tumors contain only a small fraction of cycling cells, Liau et al. used receptor tyrosine kinase inhibitors to induce a similar, slowly cycling quiescent state within a GSC population. This change from proliferation to quiescence was accompanied by changes in H3K27ac and H3K27me3 marks, with H3K27ac-associated motifs specific to the quiescent population being marked with H3K27me3 in the untreated/RTK-naïve population, and motifs related to neural stem cell development becoming enriched in H3K27ac marks in the quiescent population (36). Separate research integrated this mark of active enhancer regions with gene expression and DNA methylation data to define the enhancer landscape within GBM, finding many of the concordant loci located at genes with important functions in stem cell maintenance, such as *SOX2*, *EGFR*, *POU3F2*, and *SALL3*. Further profiling of the H3K27ac landscape in primary tumor tissue samples revealed SOX2 to be a shared TF among all GBM subtypes and normal brain tissue, while POU3F2 was preferentially found in proneural tumor samples (201). Mapping of enhancer regions from H3K27ac ChIP-seq data to high resolution fetal brain Hi-C data identified 116 enhancer-promoter pairs with significant contact frequency, corresponding to 96 total genes of which 17 were differentially expressed in GBM as compared to lower grade glioma and pilocytic astrocytoma. This list included ANXA2R, which encodes the receptor for annexin 2, a gene overexpressed in GBM and other malignancies, which is thought to contribute to cellular migration and growth (202, 203). Similar research investigating broader changes in histone lysine PTMs upon GSC differentiation observed alterations in the active enhancer regions, finding regions with both H3K27ac and H3K4me1 modifications in GSCs losing the H3K27ac modification upon differentiation, as well as increases in larger (3-50 kb) domains containing repressive H3K9me3 and H3K27me3 modifications (116). Unsurprisingly, in addition to correlations with varying degrees of GSC differentiation, there are correlations between transcriptional state and promoter histone PTMs. By superimposing paired multiplexed single cell reduced representation bisulfite sequencing and scRNA-seq onto existing ChIP-seq data, Chaligne et al. found connections between these three modalities, specifically that hypomethylated promoters in astrocyte-like and mesenchymal-like cells primarily contained H3K4me3, H3K27ac, and H3K36me3 modifications, which are associated with active transcription (204). Similarly, hypomethylated promoters in neural progenitor-like and oligodendrocyte progenitor-like cells primarily had H3K4me3 and H3K27me3 (bivalent) modifications, recapitulating the observed change from bivalent to single histone modifications during differentiation given that progenitor-like cells are less terminally-differentiated than MES- and AC-like cells (35, 204). As methods to simultaneously profile histone PTMs and gene expression at single-cell resolution become more accessible, our understanding of the correlations between the epigenetic and transcriptional landscape of GBM will increase, allowing us to better grasp the interplay between individual histone modifications, and histone modifications and downstream gene expression.

## 5 Challenges and knowledge gaps

Work on the role of EZH2 in GBM pathophysiology has revealed the more general concept that the effects of canonical histone modifying enzymes may in fact be mediated by both histone and non-histone substrates. For instance, enrichment of H3K27me3 at the *PTEN* promoter decreased gene expression, allowing for increased AKT/mTOR signaling (185). But in contrast to a histone remodeling mechanism, EZH2 also exerts a direct activating effect on STAT3 *via* methylation of lysine residue 180 (K180) (19). As highlighted by these EZH2-dependent mechanisms, there is a comparative lack of clarity surrounding the mechanism of action of many histone modifying enzymes in GBM pathophysiology. Furthermore, many of the studies that modulate the expression of individual histone modifying enzymes in GBM have used phenotypic changes as an endpoint. This results in ambiguity as to the effector(s) of these phenotypes, as they could be the result of either genomic and transcriptional dysregulation, or alterations in the function of non-histone substrates. Incorporating epigenomic profiling methods such as ChIPseq, CUT&RUN, CUT&Tag, Hi-C, and their single-cell variants would allow observation of the genomic consequences of these alterations, whereas coimmunoprecipitation, affinity purification with mass spectrometry, and similar protein-based assays could point to similar implications of altering histone PTM enzymes on non-histone substrates. Additionally, the degree to which established immortalized GBM cell lines replicate the transcriptional landscape of primary tumors has been brought into question, and recapitulating the effects of single or multi-target inhibition in multiple model systems as well as both *in vitro* and *in vivo* has become increasingly important, as previous screening between these environments has showcased a vast difference in environment-specific gene dependencies (189, 205, 206). While there have been several studies investigating the impact of single-target perturbations in GBM, many of these have relied on immortalized cell line cultures *in vitro*. This presents both a limitation of the existing work as well as an opportunity for future research in model systems and environments which better mimic that of GBM. In recent years, organoid-based model systems and embryonic stem cell-derived model systems have been validated in their ability to reflect characteristics of GBM biology (207, 208). Application of epigenetic and biochemical profiling techniques to these model systems, as well as model systems mimicking the current standard of care or *in vivo* systems could help illuminate the functional dependency of tumor cells on specific epigenetic states or enzymatic actions.

Perturbations at the DNA or RNA levels and small molecule inhibitors are powerful tools for unraveling the biological function of a gene product and have been used extensively to elucidate the role of histone modifying enzymes in GBM. Inhibition of these enzymes also holds promise in clinical therapies when found to preferentially target cancer-dependent pathways. Most human clinical trials have focused on the use of HDAC inhibitors in GBM and a recent systematic review was published supporting the use of valproic acid to increase overall survival in GBM, although prospective randomized control trials are needed to confirm these findings (209). In contrast to valproic acid, vorinostat and panobinostat have demonstrated less promising data with vorinostat leading to toxicities or failing to show benefit, and similar lack of benefit in panobinostat (85). Another challenge for HDACi trials has been difficulty with recruiting patients. It is likely that the anti-epileptic role of valproic acid has increased its use and subsequent study in glioblastoma patients. Outside of histone acetylation, there has been little clinical investigation of methylation inhibitors with a currently recruiting phase II clinical trial for the EZH2 inhibitor EPZ6438, but in pediatric gliomas. An active, but no longer recruiting phase I clinical trial is investigation PRT811, a PRMT5 inhibitor, but again without a glioblastoma focus as the trial is treating all high grade gliomas (clinicaltrials.gov). A challenge in using some of the drugs listed in this review is an inability to penetrate the BBB. This challenge can be better managed moving forward with the emergence of BBB modulating therapies such as laser interstitial thermal therapy and focused ultrasound (210, 211).

As inhibitors of histone modifying enzymes are being translated to the clinical setting, it is imperative to understand the role that these inhibitors play not just on the cancer cells but also on the tumor microenvironment, including blood and lymph vessels and tumor infiltrating immune cells. Thus, although the use of immunodeficient mice to mimic an *in vivo* environment has improved our understanding of human cancer biology, these environments lack the adaptive immune component of the human tumor microenvironment. As the number of successful immunotherapies across human malignancies grows and with increasing appreciation for the role of adaptive and innate immunity in GBM treatment resistance, the need for more complex models is needed. Multiple syngeneic mouse tumor lines have been developed to model GBM in the setting of an intact immune system. Due to the differences between human and mouse tumor biology and it is imperative that newly developed mouse tumor cell lines recapitulate the starkly immunosuppressive microenvironment of glioblastoma to ensure clinical utility. GL261 is an examples of a syngeneic mouse tumor line that possesses significant mutation burden and expresses elevated MHC1 levels, leading to a favorable immune response that arguably does not capture the full complexity of human disease (212). In contrast, the SB28 model demonstrates a greater resistance to immune checkpoint blockade, more faithfully recapitulating human glioblastoma immune characteristics (213). A recent review highlighting the importance of diverse cellular and extracellular components that contribute to the TME found in human glioblastoma adds salience to the need for more robust pre-clinical models (214, 215). Additional examples supporting the need for immunocompetent mouse models include, as mentioned earlier, prolonged inhibition of EZH2 leading to a reversion back to a pro-growth state (141). Further work in immunocompetent models has shown the role of macrophages in supporting an immunosuppressive TME partially through immune-induced changes in DNA methylation in GSCs (216). Orthogonal work in immune cells and blood cancers has shown that HDAC inhibitors affect the immune microenvironment in multiple ways. Although HDAC inhibitors appear to downregulate the primary immune response and increase the expression of PD-L1 in cancer cells, these inhibitors can also increase the adaptive immune response (217–221). Further work is clearly needed to understand the global impact of histone modifying enzyme inhibition in the GBM tumor ecosystem.

## 6 Conclusion

Encouraging efforts across the GBM research community are increasing our understanding of the roles that histone modifying enzymes play in GBM pathophysiology. Our work here summarizes these efforts and provides a framework for improvements in the field moving forward. Given the dysregulated expression of enzymes involved in histone acetylation and methylation within GBM, it is important to understand how this aspect of epigenetics potentially influences tumorigenicity and transcriptional plasticity. Furthermore, the subtype-specific correlation of histone PTMs with gene expression and methylation, as well as the reversibility of histone PTMs in response to selective inhibition, highlight the link between these epigenetic modifications and our current understanding of transcriptional heterogeneity and plasticity in GBM. Inhibition of these numerous enzymes thus holds promise as a clinical target to improve GBM patient outcomes. Although numerous clinical trials using HDAC and HMT inhibitors are underway, there remains the need for greater efforts in understanding how altering enzymatic activity of histone PTM modifying enzymes impacts genomic architecture, non-histone substrates and their respective pathways, and the complex tumor microenvironment. Our understanding of the core molecular pathways, genetic aberrations, and transcriptional states that define GBM have progressed immensely since the clinical trials that define the current standard of care, yet these advancements have unfortunately not yet led to similar transformations in the clinic. By incorporating this existing knowledge with further studies into the targetability and pathophysiology of histone PTMs with orthogonal research on the immune microenvironment, metabolome, and neuronal and glial interactions, we can provide the best scientific foundation for the success of future clinical trials and improved care for patients with GBM.

## Author contributions

CM, TW, HY, and AHK prepared the manuscript, tables, and figures. AH guided literature search strategy. AHK oversaw the project. All authors contributed to the article and approved the submitted version.
